# Predictive Value of Heart Rate and Blood Pressure on the Prognosis of Postural Tachycardia Syndrome in Children

**DOI:** 10.3389/fped.2022.802469

**Published:** 2022-03-30

**Authors:** Shuo Wang, Runmei Zou, Hong Cai, Cheng Wang

**Affiliations:** ^1^Department of Pediatric Cardiovasology, Children's Medical Center, The Second Xiangya Hospital, Central South University, Changsha, China; ^2^Department of Neonatology, Xiangya Hospital, Central South University, Changsha, China

**Keywords:** postural tachycardia syndrome, heart rate, systolic blood pressure, rate-pressure product, children

## Abstract

**Background:**

To investigate the predictive value of heart rate (HR) and blood pressure (BP) on the prognosis of postural tachycardia syndrome (POTS) in children.

**Materials and Methods:**

53 cases of children aged 5 to 15 years who visited in the Pediatric Syncope Specialist Clinic of The Second Xiangya Hospital of Central South University for unexplained syncope or syncope precursor were diagnosed with POTS by head-up tilt test (HUTT) as the POTS group. 38 healthy children aged 5 to 16 years who underwent physical examination at the Child Health Care Clinic of the hospital in the same period were matched as controls (control group). The children with POTS were followed up after 3 months of treatment and were divided into good prognosis group (40 cases) and poor prognosis group (13 cases) according to the results of HUTT re-examination and whether the symptoms improved or not. HR and BP indicators were collected from each group at baseline and during HUTT.

**Results:**

There were 91 research subjects, of which 45 are males, with a mean age of 11.52 ± 2.13 years. (1) HR at 5 and 10 min (HR 5 and HR 10, respectively), HR difference at 5 and 10 min (HRD 5 and HRD 10, respectively), and HR and BP product at 5 and 10 min (RPP 5 and RPP 10, respectively) were greater in the POTS group than in the control group (*P* < 0.01). (2) HR 5, HR 10, HRD 5, HRD 10, and RPP 10 in children with POTS were smaller in the good prognosis group than the poor prognosis group (*P* < 0.01). (3) The area under curve was 0.925 on the four combined indicators (HR 5, HR 10, HRD 5, and HRD 10), predicting a good prognosis of POTS, sensitivity of 99.99%, and specificity of 75.00%.

**Conclusions:**

HR 5, HR 10, HRD 5, HRD 10, and RPP 10 and the four combined indicators (HR 5, HR 10, HRD 5, and HRD 10) had predictive value for the POTS prognosis in children. The predictive value of the four combined indicators for the POTS prognosis was better than that of the single HR 5, HRD 5, and RPP 10.

## Introduction

Postural tachycardia syndrome (POTS) is one of the common hemodynamic types of neurally mediated syncope (NMS) in children, characterized by an excessive increase in heart rate (HR) during sudden changes in body position, which may be combined with unexplained chest tightness, dizziness or even syncope, and other symptoms of orthostatic intolerance (OI), which improve or disappear after lying down (1).

The current diagnosis of POTS is based on the standing test (ST) or the head-up tilt test (HUTT), and prognostic assessment methods for POTS have been reported in the literature (2–15). Studies have suggested that certain biological markers have predictive value for the POTS prognosis in children, such as hydrogen sulfide production in red blood cells (2), flow-mediated dilation of brachial artery (3), decrease in systolic blood pressure (SBP) or change in diastolic blood pressure when switching from a supine position to an erect position (4), body mass index (BMI) (5), baroreflex sensitivity (6), 24-h urinary sodium level (7), plasma C-type natriuretic peptide (8), midregional fragment of pro-adrenomedullin (9), postural plasma norepinephrine levels (10), salivary cortisol levels (11), HR variability (HRV) in electrocardiographic indices (12), corrected QT interval dispersion (QTcd) (13, 14), and HR and HR difference (HRD) (15). However, there is still a necessity to explore new simple non-invasive and affordable indicators to predict the POTS prognosis in children.

HR and blood pressure (BP) are the basic physiological indicators for physical function. Swai et al. (16) found that the time domain analysis of HR and HRV is a valid indicator to discriminate healthy subjects from patients with POTS by meta-analysis, but research for sensitivity and specificity is lacking. Deng et al. (4) reported that hypovolemia, elevated plasma acetylcholine levels, and abnormal vascular tone can all take effect on BP and are considered to be the main causes of POTS. The rate-pressure product (RPP) is the result of multiplying HR and SBP, which could reflect the cardiac workload and myocardial oxygen consumption, and is a good indicator for clinical assessment of human health (17), reflecting the synergistic effect of HR and SBP on the changes presented on the body, and is more meaningful than using HR or BP alone to predict cardiovascular events (18, 19). Wang et al. (20) reported that combining HR and BP parameters at different time points of HUTT can significantly improve the diagnostic efficacy of POTS. From this, we supposed that HR and BP indicators at different time points of HUTT have predictive value for the POTS prognosis. In this research, we collected HR and BP indicators at different time points of HUTT at the first visit and the follow-up HUTT with the POTS after 3 months of treatment to investigate the possible association between above indicators and the POTS prognosis and to provide a reference for the construction of a prognostic estimation model for POTS in children.

## Methods

### Participants

Clinical data were collected from April 2012 to May 2019 on 53 cases of children aged 5 to 15 years (POTS group), who attended the Pediatric Syncope Specialist Outpatient Clinic, The Second Xiangya Hospital, Central South University, for unexplained syncope or syncope precursor and were diagnosed with POTS. 38 children aged 5 to 16 years who underwent physical examination at the hospital's Specialized Pediatric Health Clinic during the same period were matched by age and gender as the control group. All research subjects completed HUTT with written informed consent signed by themselves or by their parents.

The POTS group was followed up after 3 months of treatment. According to the results of HUTT re-examination and whether the symptoms improved or not, they were divided into good prognosis group and poor prognosis group.

### HUTT

The diagnosis of POTS in this research involved only the baseline head-up tilt test (BHUT) (1). Research subjects discontinued cardiovascular active drugs that may affect autonomic function for more than five half-lives and related foods such as coffee prior to the trial. Before the experiment, 4 h of fasting and drinking were prohibited. The examination time was arranged from 8:00 a.m. to 11:00 a.m., and the environment was quiet and room temperature was 20°C−24°C. For the HUTT, the tilting device was the SHUT-100 tilt test monitoring software system, Beijing Standley Technology Co., Ltd. (Beijing, China). The subjects were kept supine on the tilt bed over 10 min, and their HR, BP, and electrocardiogram (ECG) were monitored and recorded. After recording the baseline ECG and BP of the subject in the prone position, the subject was tilted 60° with the head high and feet low position within 15 s. The HR, BP, and ECG indicators were monitored dynamically during the tilt for 10 min or at the time point of a positive response.

### Symptom Score

Symptom scores were used to assess the efficacy after treatment for POTS. The score was calculated on the basis of the frequency of clinical symptoms, the baseline score, and the follow-up score. The score includes the following clinical symptoms: dizziness, chest tightness, nausea, palpitations, headache, blurred vision, cold sweats, and syncope. The symptom scores were calculated on the basis of the following criteria: 0, no symptoms; 1, less than one time per month on average; 2, two to four times per month on average; 3, two to seven times per week on average; and 4, more than one time per day on average. The final score is the sum of each score (14, 15).

### Inclusion and Exclusion Criteria

Inclusion criteria are as follow: (1) children under 18 years old; (2) OI symptoms such as dizziness, headache, weakness, blurred vision, chest tightness, palpitations, hand tremor, limited movement in an upright position, and even syncope; and (3) HUTT (1): Supine HR is normal, and, during the initial 10 min of HUTT or the ST, HR increases ≥40 bpm or is ≥130 bpm (in children 6~12 years old) or ≥125 bpm (in adolescents 12~18 years old), without orthostatic hypotension (BP decrease >20/10 mmHg).

Exclusion criteria are as follows: excluding patients with organic cardiopulmonary diseases, neurogenic diseases, immunological diseases, and metabolic and endocrine diseases causing syncope or syncope precursor symptoms, and non-pharmacological treatments for POTS were effective.

### POTS Treatment Methods

In the condition that non-pharmacological treatment (health education, autonomic nervous function exercise, and increase the intake of water and salt) is ineffective (1), the combination of metoprolol 1 mg/(kg·d) was administered orally in two divided doses for a period of 3 months.

### Prognosis of POTS

If the clinical symptom improved and the HUTT was not consistent with the criteria for POTS, then the good prognosis group was considered. If the clinical symptom did not improve and the HUTT still consistent with the criteria for POTS, then the poor prognosis group was considered.

### Measurement Indicators

HR of HUTT at 0, 5, and 10 min (HR 0, HR 5, and HR 10, respectively); HRD between HR 0, HR 5, and HR 10 (HRD 0, HRD 5, and HRD 10, respectively); SBP of HUTT at 0, 5, and 10 min (SBP 0, SBP 5, and SBP 10, respectively); RPP of HUTT at 0, 5, and 10 min (RPP 0, RPP 5, and RPP 10, respectively).

RPP (bpm·mmHg) = HR (bpm) × SBP (mmHg).

### Statistical Analysis

SPSS statistical software (version number: 25.0, IBM Corp, Armonk, New York, USA) and MedCalc statistical software (version number: 19.0.7) were used to statistically analyze the data. Data with normally distributed were expressed as mean ± SD and count data were expressed as frequency (rate). Statistical analysis was performed by *t*-test and χ^2^ test. The sensitivity and specificity of the predictive value for the predictors were evaluated according to the receiver operating characteristic (ROC) curve, and the area under the curve (AUC) was used to indicate the predictive ability of the predictors: 0.5 ≤ AUC <0.7 means low predictive ability; 0.7 ≤ AUC <0.9 means moderate predictive ability; and AUC ≥ 0.9 means good predictive ability. When the Youden index was the largest, its sensitivity and specificity reached the best, and this cutoff point was selected as the boundary value of the predictive index. A difference was considered statistically significant at *P* < 0.05 (two-sided).

## Results

### Demographic Characteristics

A total of 91 children were enrolled in this research, which consisted of 53 cases in the POTS group (26 males and 27 females, mean age 11.83 ± 1.98 years) and 38 cases in the control group (19 males and 19 females, mean age 11.08 ± 2.28 years). The POTS group was divided into 40 cases with good prognosis (23 males and 17 females, mean age 12.38 ± 1.94 years) and 13 cases with poor prognosis (3 males and 10 females, mean age 11.65 ± 1.98 years) according to the results of HUTT re-examination and whether the symptoms improved or not.

The height of the POTS group was higher than the control group (*P* < 0.05). No statistically significant differences were seen between the POTS and control groups in gender, age, and weight (*P* > 0.05). No statistically significant differences were seen between good prognosis group and poor prognosis group in gender, age, height, and weight (*P* > 0.05) (Tables 1, 2).

**Table 1:** Comparison of general information for control group and POTS group (Mean ± SD).

**Characteristics**	**Male/Female**	**Age (years)**	**Height (cm)**	**Weight (kg)**
Control (*n* = 38)	19/19	11.08 ± 2.28	143.57 ± 14.80	37.55 ± 11.92
POTS (*n* = 53)	26/27	11.83 ± 1.98	153.30 ± 13.08	41.64 ± 9.75
t/χ^2^	0.008	−1.675	−3.314	−1.797
*P*-Value	0.929	0.097	0.001	0.076

*POTS, postural tachycardia syndrome*.

**Table 2:** Comparison of general information for good prognosis group and poor prognosis group (Mean ± SD).

**Characteristics**	**Male/Female**	**Age (years)**	**Height (cm)**	**Weight (kg)**
Good prognosis (*n* = 40)	23/17	12.38 ± 1.94	157.62 ± 13.35	44.15 ± 8.76
Poor prognosis (*n* = 13)	3/10	11.65 ± 1.98	151.90 ± 12.85	40.83 ± 10.02
t/χ^2^	3.377	−1.167	−1.381	−1.071
*P*-Value	0.066	0.249	0.173	0.289

*POTS, postural tachycardia syndrome*.

### Symptom Score

The baseline score was 2.68 ± 0.85 and the follow-up score (3 months later) was 1.02 ± 0.31, with the follow-up score significantly lower than the baseline score (*P* < 0.01). The score of the good prognosis group was 0.95 ± 0.22, the score of the poor prognosis group was 1.23 ± 0.44 (*P* < 0.05).

### Comparison of HUTT Indicators for Different Groups

HR 5, HR 10, HRD 5, HRD 10, RPP 5, and RPP 10 were larger in the POTS group than in the control group (*P* < 0.01). HR 0, SBP 0, SBP 5, SBP 10, and RPP 0 did not show statistically significant differences between POTS and control groups (*P* > 0.05). HR 5, HR 10, HRD 5, HRD 10, and RPP 10 in the good prognosis group were lower than the poor prognosis group (*P* < 0.01). HR 0, SBP 0, SBP 5, SBP 10, RPP 0, and RPP 5 did not show statistical differences between the good prognosis group and the poor prognosis group (*P* > 0.05) (Tables 3, 4).

**Table 3:** Comparison of HUTT parameters for control group and POTS group (Mean ± SD).

**Characteristics**	**HR 0**	**HR 5**	**HR 10**	**HRD 5**	**HRD 10**	**SBP 0**	**SBP 5**	**SBP 10**	**RPP 0**	**RPP 5**	**RPP 10**
	**(bpm)**	**(bpm)**	**(bpm)**	**(bpm)**	**(bpm)**	**(mmHg)**	**(mmHg)**	**(mmHg)**	**(bpm·mmHg)**	**(bpm·mmHg)**	**(bpm·mmHg)**
Control (*n* = 38)	77.68 ± 10.37	95.79 ± 13.89	96.05 ± 12.43	18.11 ± 10.44	18.37 ± 10.53	106.79 ± 12.54	107.79 ± 10.46	108.97 ± 9.66	8335.47 ± 1758.30	10371.42 ± 1910.20	10523.18 ± 1771.48
POTS (*n* = 53)	76.15 ± 12.66	102.42 ± 15.18	102.34 ± 16.77	26.26 ± 12.22	26.19 ± 14.97	110.30 ± 10.22	112.42 ± 11.69	111.98 ± 11.39	8409.96 ± 1715.90	11564.32 ± 2411.28	11446.49 ± 2549.37
*t*	0.613	−2.127	−2.054	−3.335	−2.925	−1.470	−1.944	−1.321	−2.202	−2.253	−2.038
*P-*Value	0.541	0.036	0.043	0.001	0.004	0.145	0.055	0.190	0.840	0.013	0.045

*HUTT, head-up tilt test; POTS, postural tachycardia syndrome; HR, heart rate; SBP, systolic blood pressure; RPP, rate-pressure product*.

**Table 4:** Comparison of HUTT parameters for good prognosis group and poor prognosis group (Mean ± SD).

**Characteristics**	**HR 0**	**HR 5**	**HR 10**	**HRD 5**	**HRD 10**	**SBP 0**	**SBP 5**	**SBP 10**	**RPP 0**	**RPP 5**	**RPP 10**
	**(bpm)**	**(bpm)**	**(bpm)**	**(bpm)**	**(bpm)**	**(mmHg)**	**(mmHg)**	**(mmHg)**	**(bpm·mmHg)**	**(bpm·mmHg)**	**(bpm·mmHg)**
Good prognosis (*n* = 40)	75.73 ± 9.93	98.73 ± 12.43	96.90 ± 13.96	23.00 ± 9.99	21.18 ± 11.66	109.08 ± 9.84	112.40 ± 11.31	112.10 ± 10.73	8307.78 ± 1250.60	11125.45 ± 1952.35	10819.58 ± 2144.26
Poor prognosis (*n* = 13)	77.46 ± 19.29	113.77 ± 17.65	119.08 ± 13.52	37.00 ± 13.67	41.58 ± 14.29	111.85 ± 11.58	112.46 ± 13.29	111.62 ± 13.72	8724.38 ± 2744.77	12914.69 ± 3192.12	13375.46 ± 2807.01
*t*	−0.311	−3.406	−5.012	−3.836	−5.261	−0.624	−0.016	−0.132	−0.530	−1.908	−3.455
*P-*Value	0.760	0.001	0.000	0.000	0.000	0.536	0.987	0.896	0.605	0.076	0.001

*HUTT, head-up tilt test; POTS, postural tachycardia syndrome; HR, heart rate; SBP, systolic blood pressure; RPP, rate-pressure product*.

### Comparison of the Predictive Value of Each Indicator for the POTS Prognosis

HR 5, HR 10, HRD 5, HRD 10, and RPP 10, the four combined indicators (HR 5, HR 10, HRD 5, and HRD 10), and the five combined indicators (HR 5, HR 10, HRD 5, HRD 10, and RPP10) all had a good predictive value for the POTS prognosis (*P* < 0.01).

**Figure 1:**
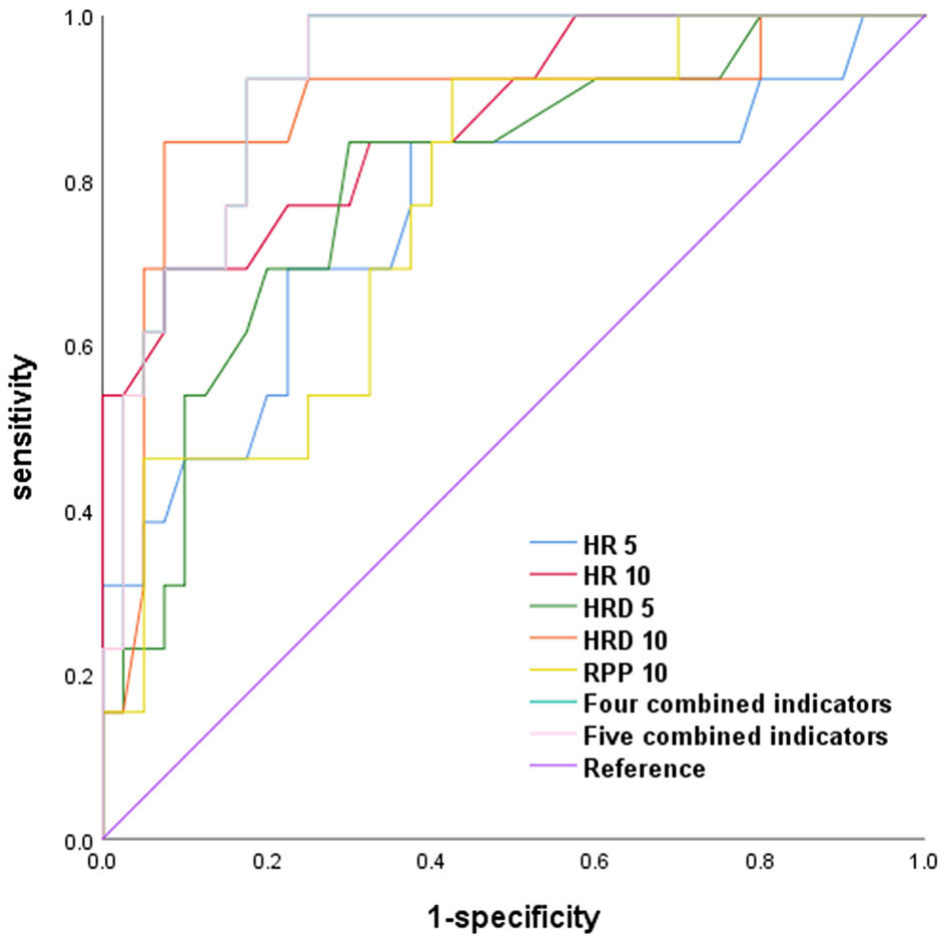
ROC comparison of HR, HRD, RPP, and combined indicators to predict the POTS prognosis.

The AUC of the four combined indicators (HR 5, HR 10, HRD 5, and HRD 10) predicting the POTS prognosis was larger than that of HR 5, HRD 5, and RPP 10 (Z = 2.026, 2.045, and 2.696, respectively; *P* = 0.043, 0.041, and 0.007), which suggests that the predictive value of the four combined indicators was better than that of HR 5, HRD 5, and RPP 10, and the sensitivity and specificity were 99.99 and 75.00%, respectively. However, the predictive value of the five combined indicators (HR 5, HR 10, HRD 5, HRD 10, and RPP 10) was not better than that of the four combined indicators, suggesting that RPP 10 was inappropriate to be added in the combined indicators to predict the POTS prognosis (Figure 1; Tables 5, 6).

**Table 5:** Comparison of ROC results for the predictive value of different indicators on the POTS' prognosis.

**Characteristics**	**AUC**	**95%CI**	***P*-Value**	**Cut-off**	**Sensitivity (%)**	**Specificity (%)**
HR 5 (bpm)	0.753	0.584–0.922	0.007	99.50	84.60	62.50
HR 10 (bpm)	0.873	0.761–0.986	0.000	115.50	69.20	92.50
HRD 5 (bpm)	0.798	0.658–0.938	0.001	27.50	84.60	70.00
HRD 10 (bpm)	0.884	0.759–0.999	0.000	36.50	84.60	92.50
RPP 5 (bpm·mmHg)	0.669	0.489–0.849	0.069	11548.50	69.20	62.50
RPP 10 (bpm·mmHg)	0.769	0.629–0.909	0.004	10988.00	92.30	57.50
Four combined indicators	0.925	0.856–0.994	0.000	-	99.99	75.00
Five combined indicators	0.925	0.856–0.994	0.000	-	99.99	75.00

*POTS, postural tachycardia syndrome; HR, heart rate; HRD, heart rate difference; RPP, rate-pressure product*.

*Four combined indicators: HR 5, HR 10, HRD 5, HRD 10*.

*Five combined indicators: HR 5, HR 10, HRD 5, HRD 10, RPP 10*.

**Table 6:** *Z*-test for comparing the predictive value of different ROCs.

**Characteristics**	**Difference in AUC**	***Z*-Value**	**95%CI**	***P-*Value**
HR 5 vs. Four combined indicators	0.172	2.026	0.006, 0.339	0.043
HR 10 vs. Four combined indicators	0.052	1.080	−0.042, 0.146	0.280
HRD 5 vs. Four combined indicators	0.127	2.045	0.005, 0.249	0.041
HRD 10 vs. Four combined indicators	0.041	0.812	−0.058, 0.141	0.417
RPP 10 vs. Four combined indicators	0.156	2.696	0.043, 0.269	0.007

*POTS, postural tachycardia syndrome; HR, heart rate; HRD, heart rate difference; RPP, rate-pressure product*.

*Four combined indicators: HR 5, HR 10, HRD 5, HRD 10*.

## Discussions

POTS prognostic estimation is very important for clinically reducing physical accidental injuries caused by OI. Certain biological markers (2–11) and electrocardiographic markers (12–15) have been found to have predictive value for the POTS prognosis. HR is modulated by autonomic nerves system and humoral regulation. BP reflects the influence of stroke output, peripheral vascular resistance, HR, elasticity of aorta wall, circulating blood volume and vascular capacity, and other comprehensive factors on the body. HR and BP are commonly and easily available physiological indicators in clinical practice, which can reflect the vital signs of physical function and also the changes of internal environment, and have been gradually applied in the diagnosis and prognosis of many diseases.

A series of changes in hemodynamics will take place while a healthy child is held in an erect position for 30 s (21–23). As the body stands erect, gravity causes blood to pool from the chest to the lower abdomen and lower limbs. This transfer of fluid directly reduces the amount of venous return and effective circulating blood volume by ~500 to 1,000 ml. As a result of reduced venous return, ventricular filling decreases, leading to a decrease in cardiac output and BP, even though mean arterial pressure remains constant (21–23), excitation of the aortic arch and carotid sinus pressure receptors causes a decrease in vagal excitability and an increase in sympathetic excitability, which, in turn, increases HR, cardiac contractility and peripheral vascular resistance to compensate for the deficit in cardiac output, maintaining BP in the normal range and avoiding syncope due to insufficient cerebral blood supply (24). The sympathetic and vagal excitability of children with POTS are in a state of imbalance, with a decrease in vagal excitability predominating, resulting in a higher HR than in healthy children. This research showed no difference in HR at baseline between children with POTS and controls, indicating that the HR of children with POTS at calm rest was essentially the same as that of healthy children. When POTS children were transferred to the erect position, the venous return decreased; the larger the HRD, the faster the HR rapidly and the shorter the ventricular diastole; and the increased peripheral resistance decreased stroke volume and cerebral perfusion, which easily induced syncope (23). Children with POTS have increased blood volume and electrolytes, increased venous filling, increased venous return and ventricular filling, and increased cardiac output and BP after increased water and salt intake (e.g., take oral rehydration salts) (25). Meanwhile, because of the increase of venous return, the sympathetic excitability decreased compared with that before treatment, the peripheral vascular resistance decreased, the positive inotropic effect of the heart was relatively weakened, and the mechanism of HR increase before treatment to compensate for the insufficient cardiac output was released, and the tachycardia symptoms were relieved in children with POTS after uprightness (26, 27). The treatment of the POTS children with metoprolol directly antagonizes catecholamines at the cellular level, inhibits adrenaline-dependent triggering activity, relatively prolongs ventricular diastole, increases cardiac output per beat, and improves blood volume and muscle sympathetic activity, resulting in a relative improvement in the occurrence of transient ischemia in the brain and a certain degree of relief of POTS symptoms (14, 26, 28). In this research, HR 5, HR 10, HRD 5, and HRD 10 were associated with the POTS prognosis. HR 5, HR 10, HRD 5, and HRD 10 were higher in POTS than controls. HR 5, HR 10, HRD 5, and HRD 10 were lower in the good prognosis group than in the poor prognosis group, which may be related to the relatively mild imbalance of neurohumoral regulation in the POTS children in the good prognosis group, and, thus, the changes of HR and HRD were lower.

HR is associated with disease prognosis. Inaguma et al. (29) reported that, in a multicenter prospective cohort study of 1,102 dialysis patients, resting HR before the first dialysis was found to be associated with all-cause mortality after starting dialysis. Patients with HR ≥ 101 bpm had significantly higher all-cause mortality than those with HR between 80 and 100 bpm, suggesting that HR may have a predictive value for disease regression. Various indicators such as HR and BP have also been reported in the literature to predict the occurrence of syncopal-like disorders (30, 31), but the predictive value of using HR or BP alone is limited (32), so this research added HRD, RPP, and combined indicators to enhance the previously limited predictive value to justify the reasonability of HR and HRD at the time of HUTT to predict the POTS prognosis.

This research found an association between RPP 10 and the POTS prognosis. RPP is the product of resting HR and SBP, which was associated with hypertensive target organ damage. As a mixed index, small changes in any of its components (e.g., a 1 mmHg increase in BP or a 1 bpm increase in HR) will produce a greatly impact to final RPP value, amplifying the impact of the physiological index and facilitating clinical observation. RPP is more suitable than the results of a single indicator (HR or BP) and provides a more comprehensive judgment of cardiovascular events compared to changes in a single indicator of HR or BP, so controlling BP and HR fluctuations can alleviate clinical symptoms in children with POTS. Verma et al. (33) found that RPP was significantly increased in patients with organic heart disease and that HR and SBP were prognostic markers of heart failure, with reduced ejection fraction in heart failure; from baseline to discharge, increased HR and SBP were associated with 30 days morbidity and mortality and increased heart failure hospitalization in patients with significantly reduced ejection fraction. Kiviniemi et al. (34) reported that post-exercise RPP values are a valid predictor of cardiac mortality in patients with coronary artery disease and type 2 diabetes, enabling risk stratification of patients with ischemic heart disease and diabetes. Gobel et al. (35) considered myocardial oxygen consumption in male patients with normal BP at rest and steady state and observed maximal exercise tolerance in angina pectoris and found that HR and RPP were good predictors reflecting myocardial oxygen consumption during exercise in patients with ischemic heart disease with normal BP. However, it has also been suggested that RPP predicts myocardial oxygen consumption in relation to species. Aksentijević et al. (36) observed that RPP values correlated with myocardial oxygen consumption in the dog or human heart, but similar results for RPP were not observed in the rat or mouse heart.

The significantly higher RPP 10 in children with POTS in this research compared to controls may be related to an increase in plasma epinephrine in erect posture, resulting in a positive correlation between RPP and plasma epinephrine (37). Children with POTS present with clinical symptoms at plasma norepinephrine levels ≥600 ng/L, and the duration of elevated plasma norepinephrine in the erect position is 30 min, much longer than the duration of elevated norepinephrine in healthy children (38). Moreover, the present research showed that RPP 10 was significantly lower in the good prognosis group compared to the poor prognosis group after POTS treatment in children, which may be due to factors such as relatively lower myocardial oxygen consumption in the good prognosis group with POTS, or relatively less stress caused by plasma epinephrine, or differences with the etiology of POTS (34, 35, 38), suggesting that RPP 10 can be used as a predictor for risk stratification of POTS.

It evidently shows that HR, HRD, and RPP and the four combined indicators (HR 5, HR 10, HRD 5, and HRD 10) have some clinical value as indicators with low clinical cost, easy access, and easy acceptance by children and their families, especially for prognostic assessment of POTS in children, and also provide new thoughts for the establishment of prognostic models of POTS in the clinic.

## Conclusions

HR, HRD, and RPP at different time points during HUTT have a high value in assessing the POTS prognosis in children. Combining a number of valuable indicators can significantly improve the effectiveness of their assessment.

## Strengths and Limitations

This research innovatively incorporated the RPP as an indicator to predict the POTS prognosis and combined valuable indicators to substantially improve the predictive value. These findings will facilitate the construction of new predictive models and deepen the understanding of the mechanisms of POTS.

The present research is a single-center, retrospective parallel case-control study with limitations such as data bias, relatively small research sample size, and a single center from one region. The applicability of the results of this research to children in other regions needs to be tested externally through a large sample, multicenter research.

## Data Availability Statement

The datasets generated for this study are available on request to the corresponding author. Requests to access these datasets should be directed to CW, wangcheng2nd@csu.edu.cn.

## Ethics Statement

The studies involving human participants were reviewed and approved by the Medical Ethical Committee, The Second Xiangya Hospital, Central South University. Written informed consent to participate in this study was provided by the participants' legal guardian/next of kin.

## Author Contributions

SW and CW conceived the research. SW, RZ, and HC collected and reviewed subjects' data. SW performed statistical analysis and drafted the manuscript. All authors contributed to its revision.

## Funding

This work was supported by 2020 Hunan Province Clinical Medical Technology Innovation Guidance Project (2020SK53405).

## Conflict of Interest

The authors declare that the research was conducted in the absence of any commercial or financial relationships that could be construed as a potential conflict of interest.

## Publisher's Note

All claims expressed in this article are solely those of the authors and do not necessarily represent those of their affiliated organizations, or those of the publisher, the editors and the reviewers. Any product that may be evaluated in this article, or claim that may be made by its manufacturer, is not guaranteed or endorsed by the publisher.
